# Proving the Correctness of Knowledge Graph Update: A Scenario From Surveillance of Adverse Childhood Experiences

**DOI:** 10.3389/fdata.2021.660101

**Published:** 2021-05-03

**Authors:** Jon Haël Brenas, Arash Shaban-Nejad

**Affiliations:** ^1^Nuffield Department of Public Health, Big Data Institute, University of Oxford, Oxford, United Kingdom; ^2^Department of Pediatrics, The University of Tennessee Health Science Center-Oak Ridge National Laboratory, Center for Biomedical Informatics, College of Medicine, Memphis, TN, United States

**Keywords:** program verification, graph transformation, cloning, merging, knowledge graph, adverse childhood experiences

## Abstract

Knowledge graphs are a modern way to store information. However, the knowledge they contain is not static. Instances of various classes may be added or deleted and the semantic relationship between elements might evolve as well. When such changes take place, a knowledge graph might become inconsistent and the knowledge it conveys meaningless. In order to ensure the consistency and coherency of dynamic knowledge graphs, we propose a method to model the transformations that a knowledge graph goes through and to prove that the new transformations do not yield inconsistencies. To do so, we express the knowledge graphs as logically decorated graphs, then we describe the transformations as algorithmic graph transformations and we use a Hoare-like verification process to prove correctness. To demonstrate the proposed method in action, we use examples from Adverse Childhood Experiences (ACEs), which is a public health crisis.

## 1. Introduction

Knowledge graphs have become ubiquitous as a framework to represent entities and the relations that connect them. Thanks to the layer of semantic information, it has become possible to add meaning to the entities and relations contained in graphs and to reason about the knowledge they contain. Because graphs contain knowledge, they are expected to change with new information added or removed depending on outside events. In a similar way, the changes in the semantic layers may cause the meaning associated with each entity to change. Through such modifications, a knowledge graph can become inconsistent with the ontology that describes it (Zhang, 2002), rendering the knowledge, and thus the graph, meaningless.

In this paper, we propose a method to tackle this issue. The proposed method aims to identify what are the transformations entailed by adding or removing a piece of information or an axiom to make sure that the knowledge graph remains consistent with the ontology. In our previous works, we used graph transformation to represent and analyze changes in knowledge-based global health surveillance systems (Brenas et al., 2017, 2018; Al-Manir et al., 2018). We represent the ontology in $C^{2}$ (Gradel et al., 1997) for convenience. The initial modification of the knowledge graphs will be assumed to be through a query language but we will represent the transformations as graph transformations and use Hoare-like verification methods (Hoare, 1969) to produce the proofs. The modifications depend only on the structure of each axiom and the action that is performed and not on the actual ontology or graph. It is thus possible to prove that they behave correctly in an abstract way that is independent of the actual knowledge graph that is modified. In particular, this means that the complexity of the verification task is independent of the size of the actual knowledge graph and only depends on the size of the specification that is proven to be correct.

To showcase how the proposed method works, we use examples coming from Adverse Childhood Experiences (ACEs), which is a major public health concern. ACEs are negative events, e.g., abuse, witnessing violence, etc, that have been linked to various negative health outcomes and risky behaviors (Felitti et al., 1998). The ACEs Ontology and knowledge graph have been described in Brenas et al. (2019a,c).

Here, we will use a clinical example. Patients or their parents are interviewed and screened for ACEs. If they suffer from some ACEs, e.g., if they are homeless or live in a place with mold, they are assigned to a social worker. If they suffered from a different set of ACEs, e.g., emotional abuse, they are assigned to a psychologist. Additionally, if they agree to be part of a study, they need to have provided a phone number or an email address and they will be paired with a caseworker. In order to avoid overworking the staff, social workers can only be assigned 10 patients and psychologists five. If the number of patients is higher than the available number of professionals, they are redirected toward a different clinic and it is agreed that a new hire is required.

In Section 2, we will introduce the formal framework that underpins our method. In Section 3, we show the applicability of this framework through some examples. Finally, in Section 4, we discuss the limitations of the method and further work.

## 2. Logic, Graph Rewriting, and Verification

Each knowledge graph is composed of a graph and an ontology that assigns meaning to its nodes and edges. In the following, we present a fairly informal and succinct introduction to our framework. We hope to manage to give readers an idea of the logical foundation of our method without spending too much time and space on information that would not be relevant to most end-users. Readers interested in a more formal and in-depth description can find it in Brenas et al. (2016a, 2018b).

In order to represent the knowledge graphs, we use logically decorated graphs where both nodes and edges are labeled with formulae that are part of the ontology language.

**Definition 2.1**. *Let C (resp. R) be a set of node labels (resp. edge labels), a logically decorated graph is a tuple (N, E, Φ_N_, Φ_E_, s, t) where N is a set of nodes, E is a set of edges, $\Phi_{N}:N\to P\left(C\right)$ is a node labeling function, $\Phi_{E}:E\to P\left(R\right)$ is an edge labeling function, s:E→N is a source function and t:E→N is a target function. The source function s and the target function t define the orientation of an edge. For instance, edge e connects node s(e) to node t(e)*.

The set C (resp. R) naturally depends on the logic. It is constructed such that C contains all “unary” (resp. “binary”) predicates of the logic and the closure under their constructors, i.e., class (resp. property) assertions.

To make our reasoning easier to understand, we describe axioms from the ontology both as first-order formulae and in a notation closer to Description Logics. In this paper, we focus on the lower spectrum of the expressivity for the axioms but the verification framework that underpins our method works with the full expressiveness of $C^{2}$ (Gradel et al., 1997), the two-variable fragment of first-order logic with counting, that can express most axioms in OWL.^1^ Informally, $C^{2}$ contains all formulas of first order logic that can be written using only two variables, as well as the counting quantifier ∃^>*n*^ and its negation ∃^≤ *n*^. As an example, ∃^>2^*x*.ϕ(*x*) can be translated in first order logic as ∃*x*_0_, *x*_1_.ϕ(*x*_0_) ∧ ϕ(*x*_1_) ∧ *x*_0_ ≠ *x*_1_, i.e., there exist at least two different *x* such that ϕ(*x*).

To explain how knowledge graphs are updated and modified, we use graph transformations. The most usual way to deal with graph transformations is by using an algebraic approach rooted in category theory (Barendregt et al., 1987). In this paper, we will use a more algorithmic approach that, we argue, is more suited to verification. In order to build transformations, we first define atomic actions that will be composed to form more elaborate actions.

**Definition 2.2**. *Let C (resp. R) be a set of node (resp. edge) labels. An elementary action, say a, may be of the following forms:*

- *a node addition add_N_(i) (resp. node deletion del_N_(i)) where i is a new node (resp. an existing node). It creates the node i. i has no incoming nor outgoing edge and it is not labeled (resp. it deletes i and all its incoming or outgoing edges)*.
- *a node label addition add_C_(i, c) (resp. node label deletion del_C_(i, c)) where i is a node and c is a label in C. It adds the label c to (resp. removes the label c from) the labeling of node i*.
- *an edge addition add_E_(e, i, j, r) (resp. edge deletion del_E_(e, i, j, r)) where e is an edge, i and j are nodes and r is an edge label in R. It adds the edge e with label r between nodes i and j (resp. removes all edges with source i and target j with label r), i.e., s(e) = i, t(e) = j and Φ_E_(e) = r (resp. ∀e′ ∈ E.s(e′)≠i or t(e′)≠j or $\Phi_{E}\left(e^{\prime }\right)\neq r$)*.

*An action α is a finite sequence of atomic actions*.

Actions are not enough to describe all the transformations we want to perform, as they require the knowledge of the exact nodes and edges that are going to be modified. In order to enable the selection of nodes that satisfy a condition, we use rewriting rules and logically decorated graph rewriting systems.

**Definition 2.3**. *A rule ρ is a pair (LHS,α) where LHS, called the left-hand side, is a logically-decorated graph and α, called the right-hand side, is an action. Rules are usually written *LHS* → α. A logically decorated graph rewriting system is a set of rules*.

**Figure 1:**
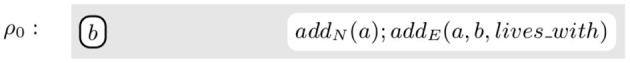
A rule that creates a new node and connects it to a previously existing one.

**Example 2.1**. *Figure 1 contains an example of rule. It selects a node, b, and creates a new node named a (add_N_(a)) and connects a to b with an edge labeled with lives_with (add_E_(a, b, lives_with))*.

A rule is applied when a match for the left-hand side is found in the knowledge graph that we want to modify. We also define strategies that describe the order in which the rules of a logically decorated graph rewriting system are to be applied.

**Definition 2.4**. *Given a logically decorated graph rewrite system GRS, a strategy is a word of the following language defined by s, where ρ is any rule in GRS:*


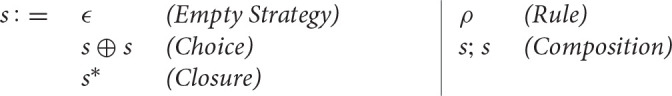


*Given two logically decorated graphs G and G′ and a strategy s, we denote by $G\Rightarrow_{s}G^{\prime }$ that it is possible to obtain G′ by applying s to G*.

Intuitively, ϵ means “do nothing,” ρ is an application of the rule, *s*⊕*s* is the application of either of the two strategies (but not both), *s*; *s* is the application composition and s^*^ applies the strategy as many times as possible.

**Example 2.2**. For instance, strategy $\rho_{0};\left(\rho_{0}^{*}⊕\rho_{1}\right)$ means “apply ρ_0_ followed by either as many applications of ρ_0_ as possible or one application of ρ_1_.”

Previously, we have shown how to use logics to label edges and nodes of graphs. We now go a little further and show how we can use logics to define specifications for the transformations we want to perform, i.e., how to define conditions that we want to be satisfied by the graph after the transformation is performed, given that it may have satisfied another (possibly identical) set of conditions initially.

**Definition 2.5 (Specification)**. *A *specificationSP* is a triple {Pre}(R,s){Post} where *Pre* (the precondition) and *Post* (the post-condition) are formulas (of a given logic), R is a graph rewriting system and *s* is a strategy*.

**Example 2.3**. Let us assume that we want a specification describing part of Example 1. $\phi_{0}\equiv \forall x.Psychologist\left(x\right)\Rightarrow \exists^{\leq 5}y.\left(Patient\left(y\right)\wedge assigned\text{\_}to\left(y,x\right)\right)$ is a formula that states that at most five patients are assigned to each psychologist. Then, $SP_{0}\equiv \left\{\phi_{0}\right\}\left(\left\{\rho_{0}\right\},\rho_{0}^{*}\right)\left\{\phi_{0}\right\}$ states that if ϕ_0_ is true, it will still be true after applying ρ_0_ as many times as possible.

**Definition 2.6 (Correctness)**. *A specification *SP* is said to be *correct* iff for all graphs *G*, *G*′ such that $G\Rightarrow_{s}G^{\prime }$ and *G* is a model of *Pre* (i.e., *Pre* is a logical consequence of the labeling of *G*), then *G*′ is a model of *Post**.

In order to prove the correctness of a specification, we use a Hoare-like approach (Hoare, 1969). The idea is that it is possible to split the transformation into elementary changes that impact the graph in a known and controlled way. In such a situation, given the post-condition that needs to be achieved, it becomes possible to generate the weakest precondition that ensures that the post-condition will be satisfied. This can then be iterated to generate the weakest precondition for the whole transformation.

**Figure 2:**
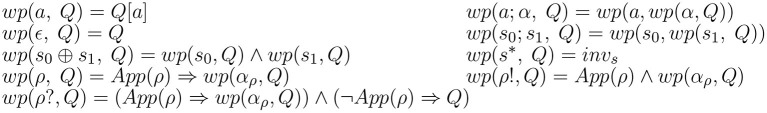
Weakest preconditions w.r.t. actions and strategies, where *a* (resp. α, α_ρ_) stands for an elementary action (resp. action, the right-hand side of a rule ρ) and *Q* is a formula.

**Figure 3:**
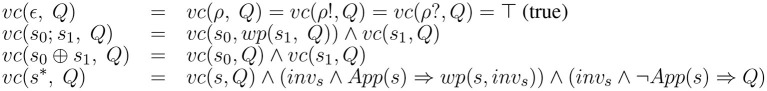
Verification conditions for strategies.

This process is achieved by two functions: the weakest-precondition *wp*(*s, Q*) and the verification condition *vc*(*s, Q*) for a strategy *s* and a post-condition *Q*. More details can be found in Brenas et al. (2016a). The definitions of these functions are given in Figures 2, 3, respectively.

The weakest preconditions and verification conditions introduce new logic constructors to deal with elementary actions called substitutions and written *Q*[*a*] where *Q* is a logic formula and *a* is an action. Intuitively, a graph *G* is a model of the formula *Q*[*a*] if and only if *G*[*a*], the graph obtained by performing action *a* on *G*, is a model of ϕ.

**Definition 2.7 (Substitutions)**. *To each elementary action *a* is associated a *substitution*, written [*a*], such that for all graphs *G* and formula ϕ, (*G* is a model of ϕ[*a*])⇔(*G*[*a*] is a model of ϕ)*.

It is worth noting that the weakest precondition of a closure, s^*^, is *inv*_*s*_, an invariant for that closure. This invariant is not part of the original specification but needs to be specified. We thus modify the notion of specification.

**Definition 2.8 (Annotated Specification)**. *An* annotated *specification SP is a triple {Pre}(R,s){Post} where Pre and Post are formulas (of a given logic), R is a graph rewriting system, s is a strategy and every closure in s is annotated with an invariant*.

**Example 2.4**. As the strategy in Example 2.3 contains a closure, we annotate it. $\left\{\phi_{0}\right\}\left(\left\{\rho_{0}\right\},\rho_{0}^{*}\left\{\phi_{0}\right\}\right)\left\{\phi_{0}\right\}$ is a possible annotated specification.

Now that the notions of the weakest precondition and the verification condition are defined, we can look back at the original problem we were trying to solve. We define a formula that represents the correctness of a specification.

**Definition 2.9 (Correctness formula)**. *We call *correctness formula* of an annotated specification SP={Pre}(R,s){Post}, the formula*:

correct(SP)=(Pre⇒wp(s,Post))∧vc(s,Post).

**Theorem 2.1 (Soundness)**. *Let SP={Pre}(R,s){Post} be an annotated specification. If *correct*(*SP*) is valid, then for all graphs *G*, *G*′ such that $G\Rightarrow_{s}G^{\prime }$, *G* is a model of *Pre* implies *G*′ is a model of *Post**.

Deciding whether a specification is correct can be translated into deciding the validity of a given formula. This is one of the main reasons why we focused on decidable logics in this section. Another possible choice is to only consider tractable logic so that verification becomes achievable in a reasonable timeframe.

The decidability of the validity problem for the logic used to label the graph is not, however, the only condition for the decidability of the correctness problem. The definitions of the weakest preconditions introduced substitutions as a new formula constructor. In order for the correctness problem to be decidable, these new constructs must be expressible in the logic, i.e., the logic must be closed under substitutions. In the following, we will be using $C^{2}$.

**Theorem 2.2**. *(Brenas et al., 2016a) $C^{2}$ is closed under substitutions*.

For all the logics that are closed under substitution, the proof consists in a set of rewrite rules that conserve the interpretation. For instance, given *C*_0_ an atomic concept, σ a substitution, *i* and *j* individuals and π_0_ a program:

- ⊤σ ↝ ⊤
- *C*_0_[*add*_*C*_(*C*_0_, *i*)] ↝ *C*_0_∨{*i*}
- (∃*r*_0_.*C*)[*del*_*E*_(*i, j, r*_0_)] ↝ (¬{*i*} ∧ ∃*r*_0_.(*C*[*del*_*E*_(*i, j, r*_0_)]))∨(∃*r*_0_.(¬{*j*} ∧ *C*[*del*_*E*_(*i, j, r*_0_)]))

Another possible problem is that the logic needs to be able to express the existence (and absence) of a match. First-order logic can express *App*(ρ) by using an existential variable for every node of the left-hand side of the rule ρ. This is not possible in the other types of logics we considered as they do not allow to define an unlimited number of variables. There is thus a limitation on what can appear on the left-hand side of the rules.

## 3. Application

When adding or removing knowledge from a knowledge graph, there is a risk to harm its consistency with the ontology that describes its underlying structure. As a result, additional actions may be needed before making any change to a knowledge graph. In this section, we present some of the transformations that can be enacted to make an update. We will then prove that the specifications resulting from the axioms and the transformations under consideration are correct and, thus, the update can take place.

To show some examples, we use the Adverse Childhood Experiences Ontology (ACESO) (Brenas et al., 2019a) as the source for the axioms. The systematic study of adverse childhood experiences and their health outcomes is recent and new data and knowledge are routinely added in this field. As a result, both the knowledge graphs and the associated ontology are likely to change frequently.

**Example 3.1**. *Let us assume that the change that we want to perform is the addition of a new node, pa, which is labeled with PhysicalAbuse. This modification would be equivalent to performing the action a_0_ ≡ add_N_(pa);add_C_(pa, PhysicalAbuse). ACESO contains the axiom PhysicalAbuse ⊆ Abuse that can be translated in $C^{2}$ as ∀x.PhysicalAbuse(x)⇒Abuse(x). Performing a_0_ would make the knowledge graph inconsistent with the ontology as, if x = pa, PhysicalAbuse(x) ∧ ¬Abuse(x) is true. Instead, the transformation that is actually performed is a_1_ ≡ add_N_(pa);add_C_(pa, PhysicalAbuse);add_C_(pa, Abuse). In that case, the correctness formula is (∀x.PhysicalAbuse(x)⇒Abuse(x))⇒(∀x.(PhysicalAbuse(x)∨x = pa)⇒(Abuse(x)∨x = pa)) which is obviously valid. The knowledge graph is thus still consistent with the ontology*.

As it is possible to modify the knowledge graph by adding information contained in new nodes, it is also possible to add new edges. In the previous example, only the new node, *pa*, was really affected by the modification of the knowledge graph and thus a simple action was enough to update the knowledge graph to preserve consistency. The next example, on the other hand, requires a rule to be applied.

**Figure 4:**
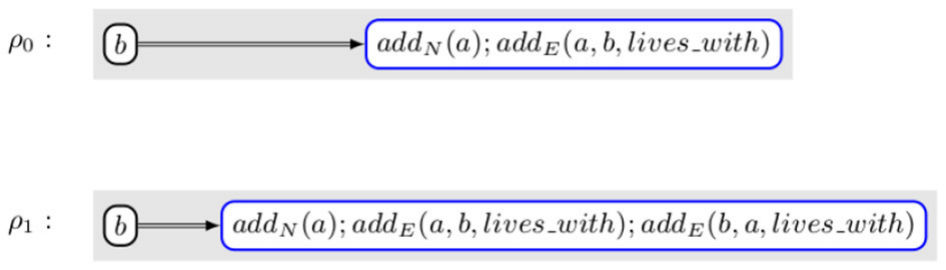
Rules creating a new node *a* and connecting it via property *lives*_*with* to the node *b*.

**Example 3.2**. *Let us assume that the change that we want to perform is the creation of a new node, a, and a new edge, e, linking a to the already existing node b with an edge labeled lives_with. This modification would be equivalent to applying Rule ρ_0_ shown in Figure 4. ACESO contains the axiom Symmetric(lives_with) that can be translated in $C^{2}$ as ∀x, y.lives_with(x, y)⇒lives_with(y, x). Applying ρ_0_ would make the knowledge graph inconsistent with the ontology as, if x = a and y = b, lives_with(x, y) ∧ ¬lives_with(y, x) is true. Instead, the transformation that is actually performed is the application of rule ρ_1_ shown in Figure 4. In that case, the correctness formula is (∀x, y.lives_with(x, y)⇒lives_with(y, x))⇒(∀x, y.(lives_with(x, y)∨(x = a* ∧ *y = b)∨(x = b* ∧ *y = a))⇒(lives_with(y, x) ∨ (x = b* ∧ *y = a)∨(x = a* ∧ *y = b)) which is obviously valid*.

**Figure 5:**
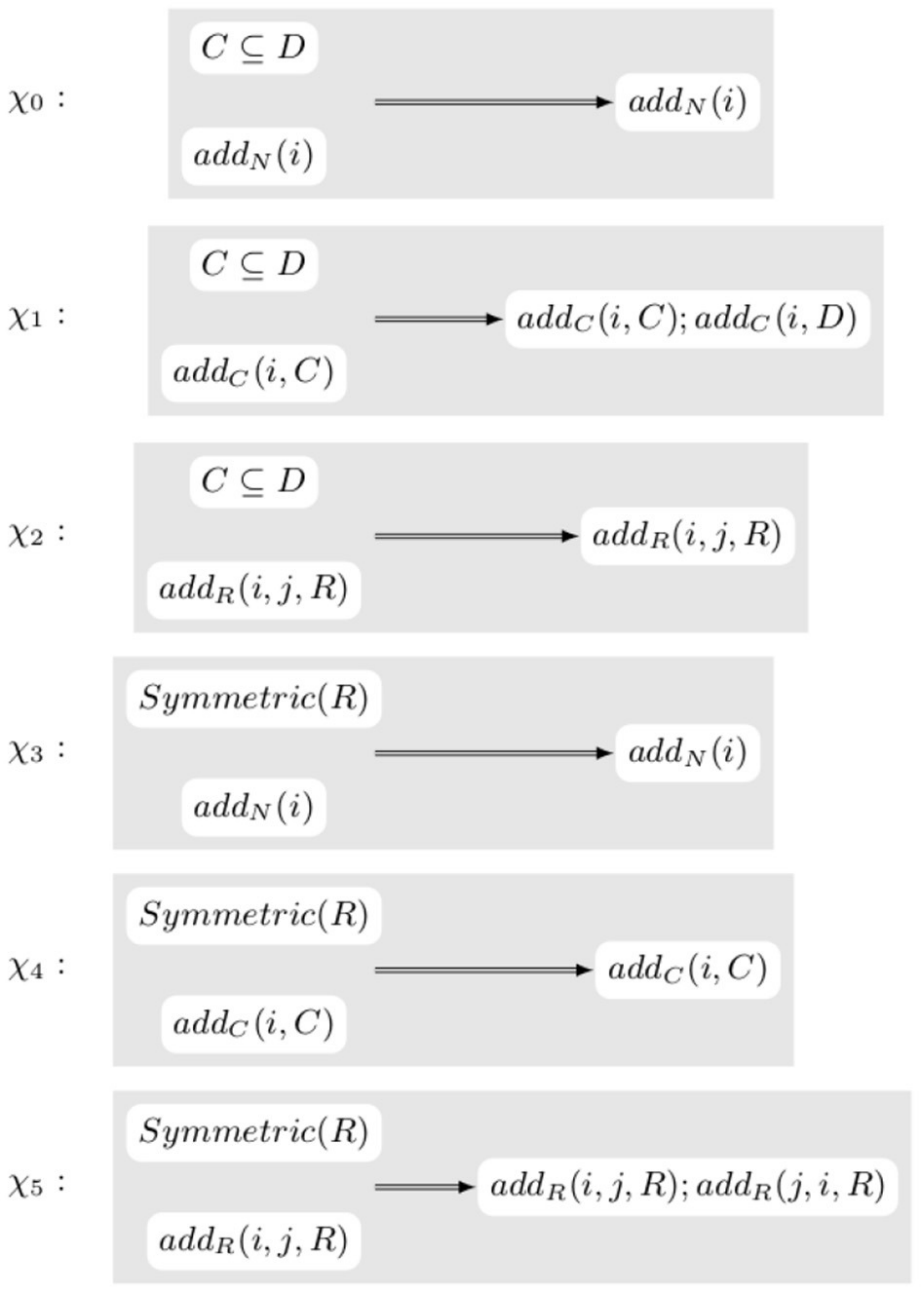
Rules rewriting transformations depending on the form of the axioms in the ontology.

From the previous two examples, one can observe how the transformations and the axioms interact. As a result, it is possible to define a system of transformation rewriting rules that, given the intended action and the form of an axiom, generates a new transformation of the knowledge graph that can be proved correct independently of the actual knowledge graph and ontology. For instance, the previous examples show that when in presence of an axiom of the form *C* ⊆ *D* where *C* and *D* are unary predicates (i.e., classes in OWL Lite), an elementary action *add*_*C*_(*i, C*) should be replaced with the action *add*_*N*_(*i, C*);*add*_*N*_(*i, D*) while, in presence of an axiom of the form *Symmetric*(*R*) where *R* is a binary predicate (i.e., a property in OWL Lite), an elementary action *add*_*N*_(*i*) should be left unchanged. Figure 5 shows these two rules plus additional ones.

**Figure 6:**
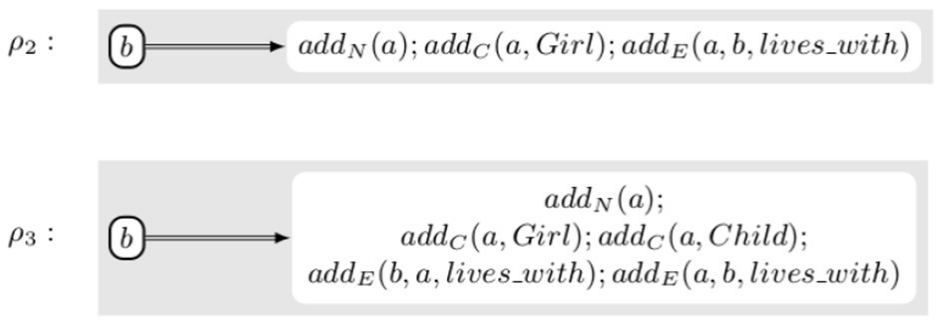
Rules inserting the knowledge that Alice, who did not exist in the knowledge base, is a girl who lives with Bob.

**Example 3.3**. *Let us now combine the previous two examples. We assume that we want to add a new person Alice, represented by the new node a, to the knowledge graph. We additionally want to insert the knowledge that Alice is a girl and that she lives with Bob, represented by the already existing node b. The initial rule that would be applied is rule ρ_2_ from Figure 6. Provided that the ontology contains the axioms Girl ⊆ Child and Symmetric(lives_with), all six rules in Figure 5 can be applied. The order in which they are applied does not change the final result, i.e., the rewriting system is convergent, and the final result is Rule ρ_3_ in Figure 6. As it is possible to prove that each one of the transformation rewrite rules is correct, their combination is correct as well*.

One of the key advantages of using abstract rules to represent the modification of the transformations is that it becomes possible to use the same rule for multiple transformations. In actual cases, the ontology is likely to contain many more axioms and, in particular, to contain a much more developed hierarchy of classes and properties. However, assuming that the ontology contains additional axioms, e.g., *Girl* ⊆ *Female* and *Child* ⊆ *Person*, the same transformation rewrite rule χ_1_ can be applied several times introducing in the action not only *add*_*C*_(*a, Child*), as shown in Figure 6, but also, *add*_*C*_(*a, Female*) and *add*_*C*_(*a, Person*) triggered by the first application of the rule. The fact that the rules are iteratively applied does not create a risk of infinite looping provided we assume that there is a mechanism to check that the rule does not already contain the added elementary actions, given that doing the same elementary action twice yields the same result as doing it only once. Similarly, in an actual execution, the rules that do not modify the transformation, e.g., χ_0_ would not be part of the system. We only show them to make our reasoning clearer and easier to understand.

Hitherto, we have only added knowledge to the graph and not removed any. Removing knowledge is not practically much more difficult, at least as long as we keep working with less expressive axioms.

**Figure 7:**
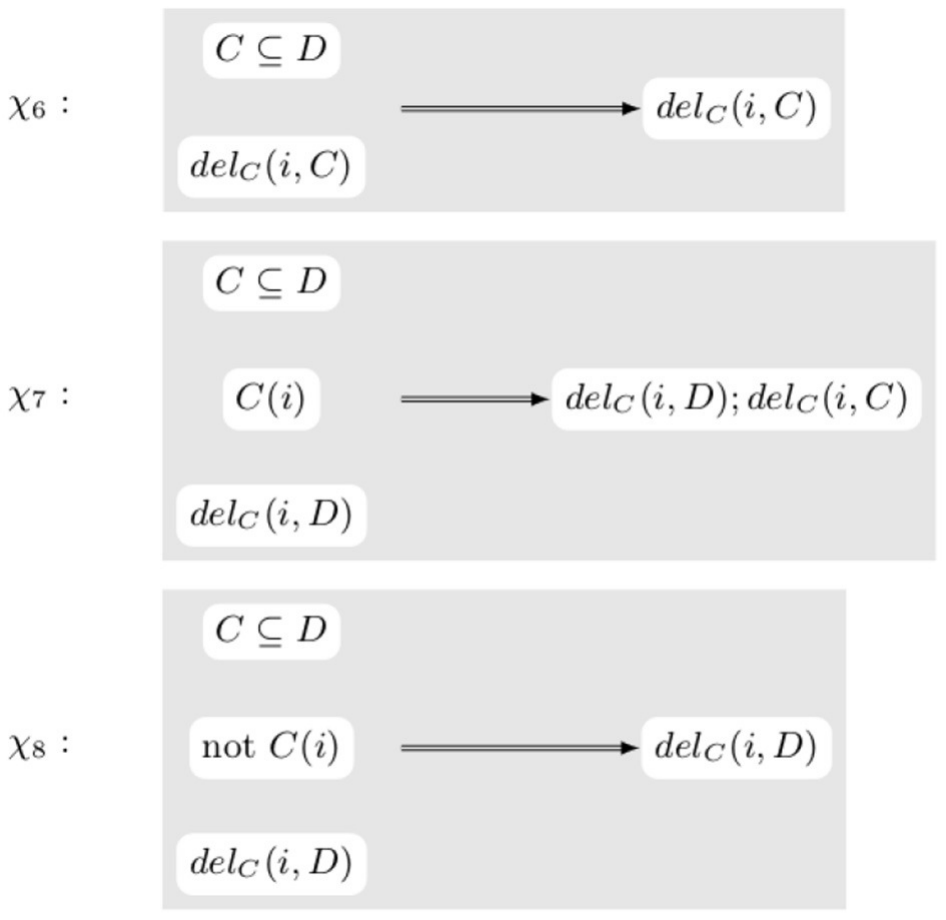
Examples of rules rewriting transformations when deleting the label of node.

**Example 3.4**. *Figure 7 contains some transformation rules dealing with node label deletion. χ_6_ is similar to the rules in Figure 5 and doesn't actually modify the transformation. On the other hand, rules χ_7_ and χ_8_ are dependent on the current content of the knowledge graph. Indeed, if C(i) is true, the axiom C ⊆ D is no longer true after the application of del_C_(i, D). Hence the rule need not only to look at the ontological part of the knowledge graph but also at the graph itself*.

Up to now, we have modified the graph by adding and changing the labeling of the knowledge graph but we have not modified the ontology itself. It is worth pointing that the choices that we made when the transformation presented a risk of inconsistency always lead us to modify the graph and not the ontology. There is, however, no reason to think that when there is a conflict between the ontology and the content of the graph, the ontology is always to be considered correct.

**Example 3.5**. *In the first example we presented, we chose when adding the fact that pa was a PhysicalAbuse knowing that PhysicalAbuse ⊆ Abuse to also add the fact that pa is an Abuse. We could also have decided to remove the axiom and the knowledge graph would have been consistent with the modified ontology*.

As the last example, we will add a new axiom to the ontology. Until now, we have only used the left-hand side of the rules to look at specific instances, either of axioms, elementary actions or individual nodes and we only, perhaps, added a new elementary action to a given rule. When modifying the ontology, a new rule is added that will modify the graph.

**Figure 8:**
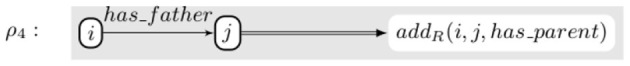
A rule that adds the fact that *has*_*father* ⊆ *has*_*parent*.

**Example 3.6**. Let us assume that we want to add the axiom has_father ⊆ has_parent to the ontology. Rule ρ_4_ presented in Figure 8 is applied to the graph with Strategy $\rho_{4}^{*}$. The correctness formula, in that case, is, after some simplification, (∀x, y.has_father(x, y)⇒has_parent(x, y))⇒(∀x, y.has_father(x, y)⇒has_parent(x, y)), an obvious tautology.

**Example 3.7**. *Let us now consider the more elaborate example of Example 1. Let us define $\phi_{0}\equiv \forall x.Psychologist\left(x\right)\Rightarrow \exists^{\leq 5}y.\left(Patient\left(y\right)\wedge assigned\text{\_}to\left(y,x\right)\right)$, $\phi_{1}\equiv \forall x.SocialWorker\left(x\right)\Rightarrow \exists^{\leq 10}y.\left(Patient\left(y\right)\wedge assigned\text{\_}to\left(y,x\right)\right)$ and ϕ_2_ ≡ ∀x.(DefSocialWorker(x)∨DefPsychologist(x)∨CaseWorker(x))⇒*

*¬(SocialWorker(x)∨Psychologist)). The precondition and the post-condition will then be ϕ ≡ ϕ_0_ ∧ ϕ_1_ ∧ ϕ_2_. ϕ_0_ states that psychologists are assigned less than five patients, ϕ_1_ states that social workers are assigned <10 patients, ϕ_2_states that default psychologists and social workers and case workers do not count as psychologists or social workers (otherwise, they might not satisfy ϕ_0_ or ϕ_1_)*.

**Figure 9:**
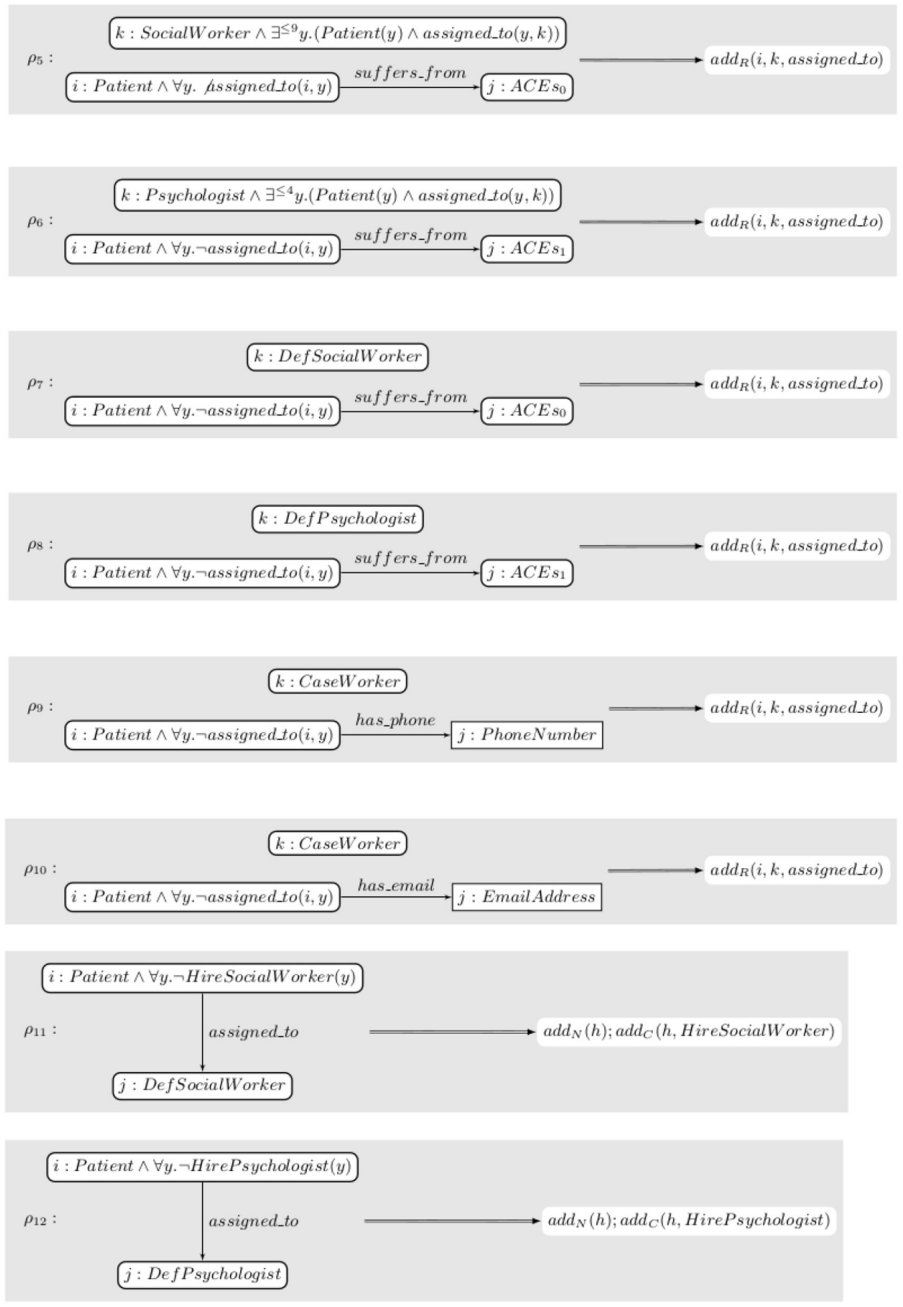
The rules used in Example 3.7.

*The set of rules R contains the rules shown in Figure 9. ρ_5_ looks for a patient suffering from a given type of ACEs, denoted by ACEs_0_, that are not assigned to anyone and for a social worker with nine or fewer patients assigned to them. It then assigns the patient to the social worker. ρ_6_ does the same for psychologists. ρ_7_ and ρ_8_ assign the (possibly) remaining patients to the default options. ρ_9_ and ρ_10_ look for patients with a phone number or an email address, respectively, and assign them to a case worker. ρ_11_ and ρ_12_ request the hiring of a social worker or a psychologist, respectively, if it had not been requested before and at least one patient is assigned to the default option*.

We chose to apply the strategy $s\equiv {\left(\rho_{5}⊕\rho_{6}\right)}^{*};{\left(\rho_{7}⊕\rho_{8}\right)}^{*};{\left(\rho_{9}⊕\rho_{10}\right)}^{n};\rho_{11}^{*};\rho_{12}^{*}$. In order to be able to perform verification, all closures need to be annotated. We annotate all of them with ϕ, that is all invariants are the same as the pre- and post-conditions. ${\left(\rho_{9}⊕\rho_{10}\right)}^{n}$ denotes n-iterations of the strategy ρ_9_⊕ρ_10_. It is a proper strategy for any given n and the actual choice of n has no impact on the correctness of the specification so we use this construct to let an unspecified number of patients register for the study. ρ_11_ and ρ_12_ can only be applied at most once each. More elaborate strategy constructors can be defined to require that strategies are applied at most once, for instance, but we did not introduce them in this paper to keep things simpler. The annotated specification is thus $s^{\prime }\equiv \left\{\phi \right\}\left(R,{\left(\rho_{5}⊕\rho_{6}\right)}^{*}\left\{\phi \right\};{\left(\rho_{7}⊕\rho_{8}\right)}^{*}\left\{\phi \right\};{\left(\rho_{9}⊕\rho_{10}\right)}^{n};\rho_{11}^{*}\left\{\phi \right\};\rho_{12}^{*}\left\{\phi \right\}\right)\left\{\phi \right\}$.

Giving the full proof that the specification is correct would be long and tedious. Instead, let us give an informal description of the proof. Neither ρ_11_ nor ρ_12_ affect any of the predicates in ϕ thus one can ignore them. ρ_9_ and ρ_10_ only modify assigned_to(x, y) for y a case worker. From ϕ_2_, one gets that being a case worker excludes the possibility of being either a social worker or a psychologist which means that if ϕ is true after ${\left(\rho_{5}⊕\rho_{6}\right)}^{*};{\left(\rho_{7}⊕\rho_{8}\right)}^{*}$, it will be true at the end of the execution. Similarly, ρ_7_ and ρ_8_ only modify the assignment to the default social worker and psychologist that, according to ϕ_2_, are not social workers or psychologists. Thus, one needs to prove that $\left\{\phi \right\}\left(R,{\left(\rho_{5}⊕\rho_{6}\right)}^{*}\left\{\phi \right\}\right)\left\{\phi \right\}$ is correct. ρ_5_ only assigns a patient to a social worker that has nine or less and thus, after the assignment, they will only have ten or less. The same argument works for psychologists albeit with a different number of edges. The specification s′ is thus correct.

## 4. Conclusion

In this paper, we demonstrated the feasibility of using graph transformations for managing changes and modifications in knowledge graphs while maintaining their consistency. We used logically decorated graphs to represent the knowledge graph, graph transformations for the modifications and a Hoare-like approach to verification to prove the correctness of the transformations.

Verification of graph transformations is an active field of computer science. Our approach is based on the method of Brenas et al. (2016b). Several other approaches exists including methods akin to model checking (Rensink et al., 2004) or the use of graph programming languages, such as GP2 (Wulandari and Plump, 2020). These methods have not been applied to knowledge graphs as far as we know.

This is only the first step toward a comprehensive solution. An important limitation to the application of our method is the fact that not all currently existing knowledge graphs are equipped with ontologies. In the absence of an ontology, the whole problem is moot as there is nothing to be consistent with. That said, it is possible to start with an empty ontology and to use our method to provably make sure that a knowledge graph becomes consistent with it by defining rules that modify it.

We have presented a few transformation rewriting rules and we are working to formulate a formal framework for their definitions. Similarly, we have only looked at the easiest of the interactions between transformations and axioms.

In particular, the rules that we have presented allow automating the process in the situation that we have presented but it is not hard to find examples where such automation is not feasible. When increasing the expressivity of the axioms, there are many situations where a non-trivial choice has to be made to decide which part of the knowledge is responsible for the inconsistency, and thus should be removed. Algorithms exist to do repairs (Bienvenu et al., 2016) that go much further than we did and integrating these algorithms into our method seems promising. That said, the verification process generates a counter model, which is a knowledge graph that invalidates the specification. This counter model can be used to decide the source of the error.

Moreover, we have only presented logics with very little expressivity in this work to make it easier to follow and to avoid problems that arise at higher expressivity. However, it has been proved that the verification that underpins this method works for the whole expressivity of $C^{2}$ (Brenas et al., 2018a). Moreover, OWL allows defining more complex axioms on properties, e.g., transitivity, that cannot be expressed in $C^{2}$ but can be expressed in other decidable logics, such as the Guarded Fragment of first-order logic (Andréka et al., 1998) for which the same results apply (Brenas et al., 2018b). Extending the expressivity to full first order logic and beyond is much more difficult as satisfiability in first-order logic is undecidable and, as a result, so is our verification process. On the other hand, it is possible to restrict the specifications to less expressive logics. Finding the conditions on the logic and the change that can be performed to reduce the complexity of the verification process is under active study. The importance of the complexity problem is somewhat reduced by the fact that the complexity of the verification task is dependent on the size of the specification and not on the size of the data it represents.

**Figure 10:**
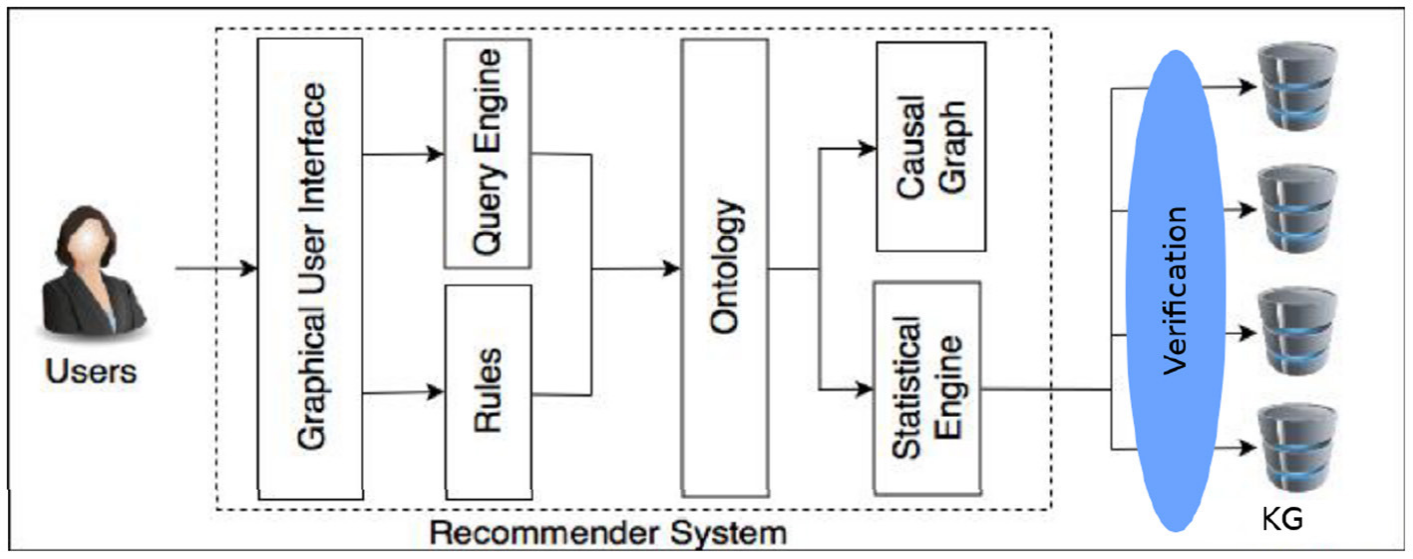
A schema of the recommender system (Brenas et al., 2019b).

Among the possible applications of the research presented in this paper is the conception of a recommender system to assist in the detection, prevention and treatment of ACEs (Brenas et al., 2019b). Figure 10 shows a schema of the recommender system's components. The recommender needs to be able to update its knowledge about the circumstances and the health of the patients in a verifiably correct manner.

## Data Availability Statement

The raw data supporting the conclusions of this article will be made available by the authors, without undue reservation.

## Author Contributions

JB and AS-N contributed equally to the research and writing of this paper.

## Conflict of Interest

The authors declare that the research was conducted in the absence of any commercial or financial relationships that could be construed as a potential conflict of interest.

## Footnotes

^1^Web Ontology Language (OWL) (accessed on January 2021).
